# Thrombophilia testing in the inpatient setting: impact of an educational intervention

**DOI:** 10.1186/s12911-019-0889-6

**Published:** 2019-08-20

**Authors:** Henry Kwang, Eric Mou, Ilana Richman, Andre Kumar, Caroline Berube, Rajani Kaimal, Neera Ahuja, Stephanie Harman, Tyler Johnson, Neil Shah, Ronald Witteles, Robert Harrington, Lisa Shieh, Jason Hom

**Affiliations:** 1 Department of Medicine, Stanford University, Stanford, CA USA; 2 Division of Hematology, Department of Medicine, Stanford University, Stanford, CA USA; 3 Department of Medicine, Yale School of Medicine, New Haven, CT USA; 4 Division of Hospital Medicine, Department of Medicine, Stanford University, 300 Pasteur Drive, HC007, Stanford, CA 94305 USA; 5 Department of Medicine, Quantitative Sciences Unit, Stanford University, Stanford, CA USA; 6 Division of Palliative Care, Department of Medicine, Stanford University, Stanford, CA USA; 7 Division of Oncology, Department of Medicine, Stanford University, Stanford, CA USA; 8 Department of Pathology, Stanford University, Stanford, CA USA; 9 Division of Cardiology, Department of Medicine, Stanford University, Stanford, CA USA

**Keywords:** Thrombophilia, High value care, Education, Inpatient

## Abstract

**Background:**

Thrombophilia testing is frequently ordered in the inpatient setting despite its limited impact on clinical decision-making and unreliable results in the setting of acute thrombosis or ongoing anticoagulation. We sought to determine the effect of an educational intervention in reducing inappropriate thrombophilia testing for hospitalized patients.

**Methods:**

During the 2014 academic year, we implemented an educational intervention with a phase implementation design for Internal Medicine interns at Stanford University Hospital. The educational session covering epidemiology, appropriate thrombophilia evaluation and clinical rationale behind these recommendations. Their ordering behavior was compared with a contemporaneous control (non-medicine and private services) and a historical control (interns from prior academic year). From the analyzed data, we determined the proportion of inappropriate thrombophilia testing of each group. Logistic generalized estimating equations were used to estimate odds ratios for inappropriate thrombophilia testing associated with the intervention.

**Results:**

Of 2151 orders included, 934 were deemed inappropriate (43.4%). The two intervention groups placed 147 orders.

A pooled analysis of ordering practices by intervention groups revealed a trend toward reduction of inappropriate ordering (*p* = 0.053). By the end of the study, the intervention groups had significantly lower rates of inappropriate testing compared to historical or contemporaneous controls.

**Conclusion:**

A brief educational intervention was associated with a trend toward reduction in inappropriate thrombophilia testing. These findings suggest that focused education on thrombophilia testing can positively impact inpatient ordering practices.

**Electronic supplementary material:**

The online version of this article (10.1186/s12911-019-0889-6) contains supplementary material, which is available to authorized users.

## Background

Venous thromboembolism (VTE) has an incidence of 1–2 per 1000 patients per year, and it is a leading cause of morbidity and mortality [1–4]. The causes of VTE are often multifactorial, with common major risk factors including surgery, trauma, immobilization, malignancy, and pregnancy [5]. A number of other risk factors, including genetic and acquired thrombophilias, can also contribute to this risk [6].

Patients with VTE often undergo laboratory evaluation for genetic or acquired thrombophilias [7]. However, recent data suggest such evaluation is often conducted inappropriately [8]. For this reason, the American Society of Hematology (ASH) “Choosing Wisely” task force recently recommended against testing for thrombophilia in adult patients with VTE occurring in the setting of transient risk factors [9].

Testing for thrombophilic tendencies may be of limited value for several reasons. First, inherited or acquired thrombophilias are very common, with detectable thrombophilia defects identified in over half of patients with known VTE [10]. Second, the presence of an abnormal test result does not necessarily predict the risk of VTE recurrence and is not helpful for risk stratification [11]. Once treatment with anticoagulation is initiated, the outcome of these patients is no different than in those without thrombophilia [10, 12–15]. Third, there have been no prospective trials evaluating anticoagulation strategies for primary prevention in patients with inherited thrombophilias and current society guidelines recommend against this [16–18]. Lastly, with the exception of the DNA-based tests, the results of thrombophilia assays can be affected by acute thrombosis or the concurrent anticoagulation regimen, leading to invalid results [12, 19].

Given the limited value of thrombophilia evaluation, hospitals and professional societies have created initiatives to lower the rate of inappropriate inpatient thrombophilia ordering. The University of Texas Southwestern Medical Center created a system in which the transfusion medicine hemostasis service would communicate with ordering physicians if thrombophilia testing did not meet established guidelines. This initiative lowered inpatient thrombophilia test ordering by 84% [20]. The University of British Columbia instituted a hard stop with a required printed request form before inpatient hereditary thrombophilia testing could be performed, and this also reduced inpatient ordering [21].

In a recently published study, we reviewed all inpatient thrombophilia ordering at Stanford Hospital over a 2-year period and found 42.7% of ordering inappropriate [6]. To address this issue, we designed an interactive educational intervention to teach house staff the reasoning behind deferring inpatient thrombophilia testing and studied whether this changed their ordering habits. As others have encouraged [22], our goal was for our educational intervention to promote long-term behavioral changes independent of practice environment and additionally encourage trainees to disseminate what they learned. In this paper, we study the impact of our educational intervention on thrombophilia test ordering practices.

## Methods

### Intervention

This was an educational intervention to improve high-value care among residents at an academic internal medicine program.

The Stanford Internal Medicine Residency program and the Division of Hospital Medicine designed an educational series to teach principles of high-value care [22]. One of these sessions during the curriculum addressed appropriate thrombophilia evaluation, epidemiology, and the clinical rationale behind the ASH recommendations.

**Fig. 1:**
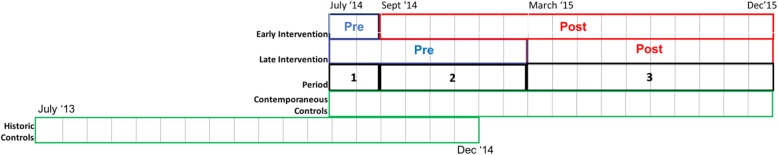
Duration and distribution of each group and interval

Internal Medicine intern physicians were randomized into two groups to receive the lecture at different times in the academic year (e.g. early-intervention group and late-intervention group). Twenty-four interns were placed in the early intervention group that received their lecture at the start of September 2014 and 25 interns were placed in the late intervention group received that their lecture at the start of March 2015 (Fig. 1). Their ordering practices were recorded from July 2014 through December 2015, thereby including the entire first year and the first 6 months of their second year. The intervention groups were not made aware that their ordering practices would be monitored. The lecture was not provided to other classes of the Internal Medicine residency, Internal Medicine attending physicians, other residency specialties, or non-teaching services.

We compared the ordering habits of the two intervention groups as a whole against two control groups. The first control was the first-year class from the prior academic year (2013), which served as a historical control. The historical control’s ordering habits were examined from their first year up to the first 6 months of their second residency year. The second control group was a contemporaneous control which included all non-intervention services during the interventional year, including third year internal medicine residents, all other residency specialties, and non-teaching private medical services. Second year internal medicine residents during the interventional observation period were excluded from the contemporaneous control group as their orders overlapped from their inclusion as the historical control in the year prior.

### Outcomes

We reviewed the following commonly ordered thrombophilia tests: factor V Leiden mutation, prothrombin G20210A mutation, antithrombin III, protein C or S activity/level, lupus anticoagulant, beta-2 glycoprotein 1 IgM/IgG antibody, anticardiolipin IgM/IgG, dilute Russell viper venom time, and JAK2 V167F mutation. We defined criteria for test appropriateness using current society guidelines and current published evidence [9, 13, 23–25]. The criteria were reviewed by a senior hematologist at our institution (CB) with specific expertise in thrombophilia and thrombosis (Tables 1 and 2).

**Table 1:** Timing and context used to determine appropriateness of testing

Assay	Acute Thrombosis	UFH	LMWH	Warfarin	DOAC	Within 2w of stopping VKA
Factor V Leiden	√	√	√	√	√	
Prothrombin gene mutation	√	√	√	√	√	
Protein C	?	?	?	X	X	X
Protein S	?	?	?	X	X	X
Antithrombin III	X	X	X	?	X	
Lupus anticoagulant	√	X	X	?	X^a^	
dRVVT	√	√	√	?	X	
Beta-2 glycoprotein 1 Ab	√	√	√	√	√	
Anti-cardiolipin Ab	√	√	√	√	√	
JAK2 mutation	√	√	√	√	√	

^a^ False Positive

**Table 2:** Criteria Used to Determine Appropriateness of Test Ordering

Inappropriate Ordering	
Patient characteristics	
Provoked VTE occurring in the setting of major transient risk factor	
Women with pregnancy-related VTE	
Patients with advanced liver disease and abnormal function	
Ordering factor V Leiden or prothrombin gene mutation in patients who have had a liver transplant (no correlation between patient’s DNA and factor status produced by the new liver)	
Testing for the MTHFR polymorphism (obsolete test, does not correlate with risk of VTE)	
Timing issues	
Ordering antithrombin III, protein C or S level during the first 3 months of anticoagulant therapy	
Ordering protein C or S levels during warfarin therapy, or within 2 weeks of stopping warfarin	
Ordering lupus anticoagulant, antithrombin III, protein C or S levels in patients on novel oral anticoagulants	
Duplicate ordering of heritable thrombophilias: factor V Leiden, prothrombin gene mutation, JAK2 V167F mutation).	
Ordering of heritable thrombophilia workups (FV Leiden, Prothrombin gene mutation, and protein C/S levels) in the inpatient setting, if fails to impact clinical management decisions.	
Appropriate Ordering	
Patients with unprovoked VTE in whom test results may impact duration or choice of anticoagulant (e.g. positive antiphospholipid antibody screening)	
Patients with VTE and multiple family members with history of VTE (higher risk of thrombophilia such as AT3 deficiency)	
Thrombosis at unusual sites (e.g. splanchnic, Budd-Chiari, renal, or cerebral venous thrombosis)	
Recurrent provoked VTE	
Screen for antiphospholipid antibody in the setting of recurrent pregnancy loss (we can define further)	
Unexplained arterial thromboses	
Ordering antiphospholipid antibody testing in the setting of arterial thrombosis (e.g. peripheral arterial events, CVA)	
Unclear Ordering	
Female patients who develop VTE on hormonal therapy	
Patients with upper extremity DVT	
Testing ordered at the patient’s request	
VTE with minor risk factor (e.g. travel-related or flight < 6-8 h, minor surgery, prolonged sitting, etc.)	
VTE in young patients with stroke and PFO	

Two independent reviewers (HK, EM) then performed manual review of electronic medical records of all patients included in the data set to determine the clinical history, timing of testing, and concurrent anti-coagulant use. The reviewers determined the appropriateness of each test ordered based on the the patient’s presentation and scored each entry as appropriate, inappropriate, or unclear based on defined criteria. Any test that was initially felt unclear by a single rater was then reviewed jointly by the study authors and only left as unclear if a conclusion still could not be made. The inter-rater correlation concordance was 0.88 for identical data sets of the first 100 entries [6].

Using the manually reviewed data set of all inpatient thrombophilia ordering, we analyzed the change in ordering behavior of the two intervention groups before and after their respective intervention over an 18 month period. This 18 month period constituted the entirety of the intervention group’s intern year as well as the first 6 months of their second residency year.

To examine the overall effect of the intervention on thrombophilia testing and to account for the potential that an educational intervention could not only decrease inappropriate ordering but also inadvertently decrease appropriate thrombophilia testing, we also recorded the total number of patients admitted during the study period to account for fluctuations in patient population. The rate of appropriate and inappropriate testing was determined per 1000 patients admitted with the assumption of a constant rate of VTE cases per 1000 patients admitted.

### Data

We examined all inpatient thrombophilia testing from June 2013 through December 2015, extracted from our hospital’s electronic medical records. The extracted data included the patient’s age, sex, medical record number, admission date, thrombophilia testing ordered, ordering provider and service, and admission diagnosis. Depending on the time of test order and the provider who ordered the test, the tests were categorized as pre or post-intervention, early or late intervention, historical or contemporaneous controls.

### Statistical analysis

To estimate the impact of the intervention on rates of inappropriate thrombophilia test ordering, three comparisons were utilized. The first was a pre-post comparison in each of the two groups of internal medicine residents that received the intervention. The second comparison looked at the pre-post change in the intervention groups compared to attendings and residents of other services, during the same time period, who did not receive the intervention (contemporaneous controls). The third comparison looked at the pre-post change in the intervention groups compared to the internal medicine residents from the prior academic year who did not get the intervention (historical controls). In all three cases, estimates of the intervention’s impact were obtained for the early and late groups combined (overall) as well as separately through stratified analysis. As patients may be seen by multiple providers and providers may see multiple patients, we considered the test ordering data to be clustered by patient and provider in a cross-classified manner. Binary logistic Generalized Estimating Equations were used to estimate the association (Odds ratio, 95% confidence interval and Wald *p*-value) between the intervention and inappropriate testing, with robust standard errors to account for the cross-classified clustering of tests by patient and provider according to the methodology of Miglioretti and Heagerty [26]. For the purposes of our study, *p* < 0.05 is considered statistically significant. Full details of the model specifications used can be found in Additional file 1.

## Results

### Ordering habits pre and post intervention

We reviewed 2584 inpatient thrombophila orders and excluded 433 (16.8%) based on pre-defined exclusion criteria (Additional file 1: Table S1). Of the 2151 qualifying orders, 934 (43.4%) were found to be inappropriate based on pre-defined inappropriateness criteria.

The two intervention group participants placed 147 orders within the 18 month observation period from July 2014 to December 2015. After their intervention in the first half of the year, the early intervention group’s ordering habits changed from 40.0% inappropriate (2/5 orders) to 24.5% inappropriate (13/53 orders) (*p* = 0.60). Similarly after their intervention in the second half of the year, the late intervention group of 25 interns changed their inappropriate ordering from 39.1% inappropriate (18/46 orders) to 23.3% inappropriate (10/43 orders) (*p* = 0.11). Individually, the two intervention groups did not demonstrate statistically significant improvement after their respective interventions. Together, the two intervention groups decreased ordering from 39.2% inappropriate (20/51 orders) to 23.0% inappropriate (23/96 orders), a finding that trended toward statistical significance (*p* = 0.053) (Table 3).

**Table 3:** Frequency and percent of inappropriate thrombophilia tests among interventional groups - pre and post intervention

Group	Pre-Intervention	Post-Intervention	*P* Value
Early Intervention	2/5 (40.0%)	13/53 (24.5%)	0.60^a^
Late Intervention	18/46 (39.1%)	10/43 (23.3%)	0.11
Combined Intervention	20/51 (39.2%)	23/96 (24.0%)	0.053

*P* values from Chi square test except where indicated

^a^ Fisher Exact test

Overall in the intervention groups, the odds of inappropriate testing post intervention were 53% less than those prior to the intervention (OR = 0.47; [95% CI: 0.14–1.61]; *p* = 0.23). When comparing the pre- and post- change for the intervention groups to that of a contemporary control group, the overall reduction in inappropriate testing was 54% (OR = 0.46; [95% CI: 0.12–1.71]; *p* = 0.25). The overall impact of the intervention was again similar when compared to the historical control from the prior year (OR = 0.42; [95% CI: 0.09, 2.06]; *p* = 0.29). While no estimates of the interventions impact achieved statistical significance (*p* < 0.05), all estimates across early and late periods intervention groups and the different control groups were consistent (Table 4).

**Table 4:** Estimated impact of intervention by intervention cohort and comparison group^a^

Intervention group	Comparison		
	Within intervention	Contemporary controls	Historical controls
	Odds Ratio (95% CI); *p*	Odds Ratio (95% CI); *p*	Odds Ratio (95% CI); *p*
Overall	0.47 (0.14, 1.61); 0.23	0.46 (0.12, 1.71); 0.25	0.42 (0.09, 2.06); 0.29
Early intervention	0.49 (0.08, 2.98); 0.44	0.46 (0.09, 2.40); 0.35	0.47 (0.08, 2.91); 0.42
Late intervention	0.47 (0.11, 2.04); 0.32	0.43 (0.10, 1.77); 0.24	0.37 (0.06, 2.35); 0.29

*CI* Confidence Interval. *p* = Wald *p*-value

^a^ All models include clustering for patient and provider

### Ordering behavior by discrete intervals

We further evaluated the chronological correlation between the intervention and ordering habits by analyzing the intervention period during 3 discrete time intervals. Interval One occured before either group received their educational sessions and spanned from July 2014 through August 2014. Interval Two ranged from September 2014 through February 2015, encompassing the time period between early and late interventions. Interval Three ranged from March 2015 through December 2015, covering the time period after both intervention groups had received education (Fig. 1).

Over the course of these three intervals, the rate of inappropriate orders in the early intervention group changed from 40.0% (Interval One) to 36.4% (Interval Two) to 16.1% (Interval Three). Despite already receiving the intervention, there was only a 3.6% improvement in ordering from 40.0 to 36.4% between the first and second interval. The rate of inappropriate orders in the late intervention group changed from 40.0% (Interval One) to 38.1% (Interval Two) to 23.3% (Interval Three) inappropriate orders. There was no significant difference in ordering habits between the two intervention groups within each interval.

**Fig. 2:**
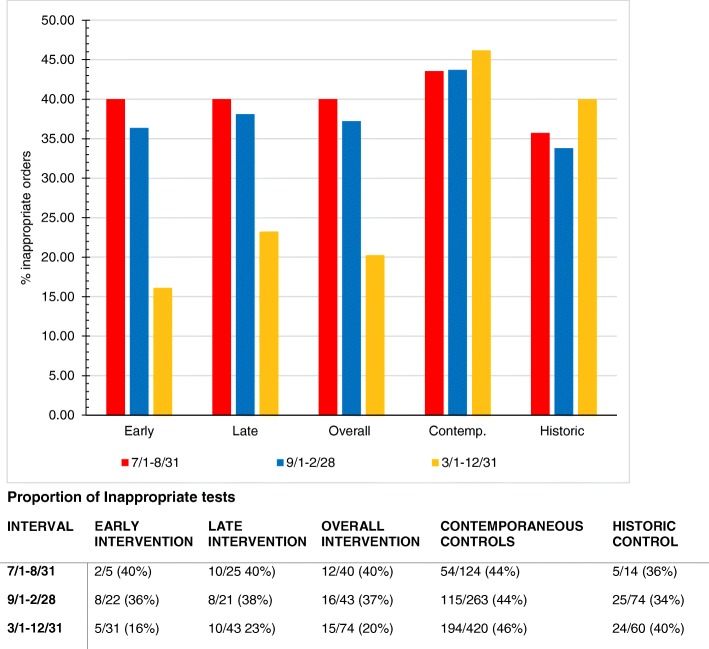
Percentage of inappropriate tests in each time interval

Combined, the two intervention groups improved from 40.0 to 37.1% to 20.3% across each interval. We also monitored ordering habits of the contemporaneous control group during these time intervals and of the historical control group during the same intervals from the previous year. Neither the historical control nor the contemporaneous control showed improvement in inappropriate ordering, with the former ordering 35.7, 33.8, 40.0% and the latter ordering 43.5, 43.7, and 46.2% over their respective time intervals. When combining our observations for the two intervention groups over the study period, the combined intervention group showed statistically significant improvement compared to both the Historical Control (*P* = 0.001) and the Contemporaneous Control groups (*P* < 0.001) (Fig. 2).

### Standardization for census

Total University Medicine patient census was utilized to further determine whether the decrease in inappropriate orders over time in the intervention groups could be explained by fluctuations in patient census. Because of the potential that an educational intervention would also inadvertently decrease appropriate thrombophilia testing, we also explored standardized appropriate testing over time. With the assumption of a constant rate of VTE per 1000 patients, we calculated the appropriate and inappropriate testing per 1000 patients admitted. There was no significant change in appropriate testing before or after intervention in either intervention group. There was no significant change in inappropriate testing in the early intervention group while the late intervention group demonstrated a statistically significant decrease in inappropriate orders per 1000 patients (*p* = 0.043) (Table 5).

**Table 5:** Tests and Confidence Interval^a^ per 1000 patients admitted

	Pre-Intervention	Post-Intervention	*P* value
Early Intervention^a^			
Inappropriate tests	3.68 (0–8.76)	3.13 (1.43–4.83)	0.832
Appropriate tests	5.51 (0–11.74)	9.63 (6.63–12.61)	0.343
Late intervention			
Inappropriate tests	8.47 (4.57–12.36)	3.89 (1.48–6.30)	0.043
Appropriate tests	13.17 (8.32–18.02)	12.84 (8.49–17.19)	0.92

^a^ Lower confidence limit restricted to 0

## Discussion

We previously performed a retrospective analysis of inpatient thrombophilia testing practices at Stanford Hospital and Clinics and demonstrated a 42.7% inappropriate ordering rate over a two-year period [6]. Thrombophilia testing is relatively costly, with the Canadian Agency for Drugs and Technologies and Health determining that routine testing for certain inherited thrombophilias after first time VTE does not have clinical utility and eliminating or reducing such testing would lead to significant cost savings [27].

Although there are certain clinical scenarios where it is appropriate to order thrombophilia testing (e.g., if antiphospholipid antibody syndrome or paroxysmal noctural hemoglubinuria is suspected), thrombophilia testing should often be avoided in hospitalized patients with unprovoked VTE because inaccuracies with testing results, cost, patient anxiety, and inappropriate prolongation of anticoagulation [28]. Furthermore, thrombophilia testing takes time and may not be available until after the patient is discharged, which could lead to unnecessary repeat testing by outpatient providers if the status of these pending tests are not communicated. Initiatives to reduce such testing have increasingly become a topic of interest across multiple heathcare systems, with efforts mostly focused on developing system-level interventions to prevent inappropriate ordering. To the best of our knowledge, our study is the first published attempt at showing how a targeted educational intervention directy impacted thrombophilia testing. Such educational interventions are important as they can promote ongoing, long-term practice pattern improvement by targeting trainees at a formative time in their careers.

The combined intervention groups showed a decrease in inappropriate thrombophilia ordering after their interventions, a result that trended toward statistical significance. Individually, the two educational intervention groups also demonstrated an overall decrease in inappropriate ordering, but this result did not reach approach statistical significance, likely due to the small sample size. Reassuringly, we determined that the rate of appropriate thrombophilia testing did not decrease during the intervention, suggesting that our efforts at discouraging inappropriate ordering did not result in excessively cautious ordering behaviors.

Notably, during Interval Two spanning from September 2014 through February 2015, the early intervention group had already received their intervention but the late intervention group had not yet received it. Despite this, both intervention groups ordered inappropriate thrombophilia tests at a similar rate that did not differ from the control arms, making it difficult to definitively show a direct impact from our intervention. It is possible that the small sample sizes analyzed for the early intervention group, especially during Interval One, are the reason we did not see a change until the third interval. With small sample sizes when dividing into time intervals, chance could play a role in these findings. Alternatively, these results could reflect the time needed for a significant culture change amongst the housestaff or attendings. Indeed, all intervention participants were second year residents who have more autonomy with regard to patient orders by Interval Three. Perhaps in this more supervisory role the participants felt more empowered to practice high-value ordering habits.

The lack of improvement in ordering habits in the contemporaneous control group suggests that institution-wide ordering practices did not change over the study period. Similarly, the historical control group did not improve ordering habits, suggesting that intern resident physicians not exposed to the education intervention did not simply improve as they progressed through their training. This is important because our intervention groups outperformed the historical and contempraneous controls at the end of the study period, suggesting a positive impact of the intervention.

### Study strengths

The educational intervention session was designed by a multidisciplinary team that included multiple specialties, and the timing and content of sessions was reviewed by our residency program leadership. Interns were randomized into their intervention groups and were not aware that their ordering patterns would be analyzed, in order to reduce the Hawthorne effect.

We performed a comprehensive review of institution-wide inpatient thrombophilia testing over 30 months to create a large sample size of thousands of entries to which we would be able to compare with the intervention groups over time. Through manual review of every patient medical record and utilization of pre-defined criteria for assessment, we thoroughly substantiated the appropriateness of each test.

### Study limitations

As we conducted a single-center study with 49 residents in the Internal Medicine intern class, we had a small intervention group sample size. Additionally, the follow-up duration was limited. We had limited power to detect small to moderately-sized effects, particularly given that thrombophilia testing is relatively uncommon. We were unable to analyze thrombophilia testing of patients with non-thrombotic conditions as there are no established guidelines.

## Conclusion

Thrombophilia work up findings obtained in the inpatient setting are often unreliable and generally do not alter clinical management. We looked at the impact of an educational intervention to reduce unnecessary thrombophilia testing among residents at an academic internal medicine program. Our findings suggest that a brief educational intervention can have a positive and sustained impact on ordering of thrombophilia testing. Prior investigators have examined a variety of promising online educational resources for trainee engagement and for continuing medical education, including social media venues such as Facebook or Twitter [29, 30]. Accordingly, future educational efforts at our institution may involve interactive, innovative online approaches. Currently, we are studying the combination of educational interventions and best practice alerts to further reduce the number of inappropriate thrombophilia tests.

## Additional file

Additional file 1: Compilation of criteria used for determining appropriateness and statistical models used for analysis. (DOCX 59 kb)

## Data Availability

The datasets analyzed during the current study are not publicly available due to inclusion of protected health information but are available from the corresponding author on reasonable request.

## Abbreviations

ASH: American Society of Hematology
VTE: Venous thromboembolism
