# Supplementary material for: Thrombophilia testing in the inpatient setting: impact of an educational intervention
Source: BMC Med Inform Decis Mak. 2019 Aug 20;19:167. doi: 10.1186/s12911-019-0889-6 (PMC6701078; doi:10.1186/s12911-019-0889-6)
Supplement: Supplementary file 1 — Compilation of criteria used for determining appropriateness and statistical models used for analysis. (DOCX 59 kb) [file 12911_2019_889_MOESM1_ESM.docx]

**Additional file 1**

**Thrombophilia Testing in the Inpatient Setting: Impact of an Educational Intervention**

**Table of contents:**

**Heading Page**

Additional Table 1: Exclusion Criteria 2

Additional Table 2: Data for each study interval and group 3

Additional Text: Pre-post comparison among the intervention groups 4

Additional Text: Contemporary and historic control comparisons 5

Additional Table 3: Comparisons and models used to estimate the impact of the intervention 6 - 7

**Table S1: Exclusion Criteria**

| Workup of isolated cytopenias |
| --- |
| Workup of new or known autoimmune diseases (e.g. SLE, rheumatoid arthritis) |
| Workup of cutaneous lesions and rashes |
| Patients with left ventricular assist devices |
| Standard workup of undifferentiated pulmonary hypertension |
| Workup of isolated elevated PTT or PT |
| Workup sent solely on suspicion of possible thrombotic event without diagnostic confirmation |
| Tests ordered with no documentation or justification |

**Table S2: Data for each study interval and group.**

| **Study Group** | **Measure** | **Overall** | **Interval 1** | **Interval 2** | **Interval 3** |
| --- | --- | --- | --- | --- | --- |
| Early Intervention Group | Providers: | 10 | 2 | 5 | 4 |
|  | Patients: | 15 | 2 | 5 | 8 |
|  | Tests: | 58 | 5 | 22 | 31 |
|  | Inappropriate: | 15 | 2 | 8 | 5 |
| Late Intervention Group | Providers: | 15 | 5 | 4 | 7 |
|  | Patients: | 19 | 5 | 4 | 10 |
|  | Tests: | 89 | 25 | 21 | 43 |
|  | Inappropriate: | 28 | 10 | 8 | 10 |
| Contemporaneous Control Group | Providers: | 93 | 17 | 42 | 56 |
|  | Patients: | 145 | 21 | 52 | 75 |
|  | Tests: | 807 | 124 | 263 | 420 |
|  | Inappropriate: | 58 | 54 | 115 | 194 |
| Historic Control Group | Providers: | 23 | 1 | 12 | 12 |
|  | Patients: | 26 | 3 | 12 | 12 |
|  | Tests: | 148 | 14 | 74 | 60 |
|  | Inappropriate: | 54 | 5 | 25 | 24 |

**Pre-post comparison among the intervention groups**

The mean model for the logistic regression to assess the impact of the intervention was specified as follows:

$$logit\left[ P\left( y_{ijk}=Inappropriate \right) \right]=\beta_{0}+\beta_{1}\times Post Intervention_{ijk}+ \beta_{2}\times Late Cohort_{ijk}$$

Where $y_{ijk}$ represents whether the *i*^th^ test for the *j*^th^ patient ordered by the *k*^th^ provider was appropriate or inappropriate. *Post intervention* is a binary indicator for the test being ordered after the medical resident received the intervention and *Late Cohort* is a binary indicator to allow for differences in average testing rates between the early and late intervention groups. Under the assumption of an independence working correlation structure to account for clustering of tests by provider and patient, three robust models were estimated as follows 1) clustering by provider, 2) clustering by patient, and 3) clustering by the combination of patient and provider, which produced three empirical covariance matrices: $V^{1}$, $V^{2}$ and $V^{3}$, respectively. This is also the general approach taken for the models that utilize contemporary or historic controls detailed below, and so for brevity we do not repeat this detail.

We obtain an estimate of the intervention effect $\tilde{\beta}_{1}$ and the standard errors associated with this and the other estimated regression coefficients in the model as $V\left( \tilde{\beta} \right)=V^{1}+V^{2}-V^{3}$. The approach we used to obtain $V\left( \tilde{\beta} \right)$ for a cross-classified GEE follows that outlined in Miglioretti and Heagerty 2007.^26^

Individual estimates for the early and late cohorts respectively, were obtained by stratifying the data and fitting the following model:

$$logit\left[ P\left( y_{ijk}=Inappropriate \right) \right]=\beta_{0}+\beta_{1}\times Post Intervention_{ijk}$$

See Additional Table 5 for a full summary of the comparisons and models used to estimate the impact of the intervention.

**Contemporary and historic control comparisons**

The mean model for the logistic regression to assess the overall impact of the intervention compared to either the contemporary control group or the historic control group was specified as follows:

$$logit\left[ P\left( y_{ijk}=Inappropriate \right) \right]=\beta_{0}+\beta_{1}\times Period 2_{ijk}+\beta_{2}\times Period 3_{ijk}+ \beta_{3}\times Early Cohort_{ijk}+ \beta_{4}\times Late Cohort_{ijk}+\beta_{5}\times Period 2_{ijk}\times Early Cohort_{ijk}+\beta_{6}\times Period 3_{ijk}\times Early Cohort_{ijk}+ \beta_{7}\times Period 2_{ijk}\times Late Cohort_{ijk}+ \beta_{8}\times Period 3_{ijk}\times Late Cohort_{ijk}$$

Here *Period 2* is a binary indicator for the time period between the early and late intervention time points and *Period 3* is a binary indicator for the period after the late intervention time point. *Early Cohort* and *Late Cohort* are binary indicators for the respective intervention groups. Based on the coefficients involved in the interaction term, the following linear combination was used to obtain an overall estimate of the impact of the intervention: $0.25\beta_{5}+0.25\beta_{6}-0.5\beta_{7}+{0.5\beta}_{8}$.

Individual estimates for the early and late cohorts respectively were obtained by stratifying the data and fitting the following model:

$$logit\left[ P\left( y_{ijk}=Inappropriate \right) \right]=\beta_{0}+\beta_{1}\times Post Intervention_{ijk}+ \beta_{2}\times Cohort_{ijk}+\beta_{3}\times Post Intervention_{ijk}\times Cohort_{ijk}$$

Where the interaction term $\beta_{3}$ provides an estimate of the interventions impact (difference between pre and post change relative to the control group). See Additional Table 2 for a full summary of the comparisons and models used to estimate the impact of the intervention

**Table S3: Comparisons and models used to estimate the impact of the intervention**

| **Model** | **Comparison** | **Model equation** |
| --- | --- | --- |
| Model 1 | 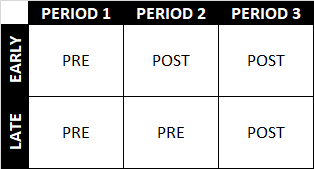 | $logit\left[ P\left( y_{ijk}=Inappropriate \right) \right]=\beta_{0}+\beta_{1}\times Post Intervention_{ijk}+ \beta_{2}\times Late Cohort_{ijk}$ |
| Model 2 | 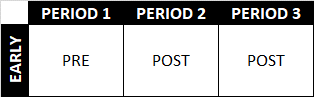 | $logit\left[ P\left( y_{ijk}=Inappropriate \right) \right]=\beta_{0}+\beta_{1}\times Post Intervention_{ijk}$ |
| Model 3 | 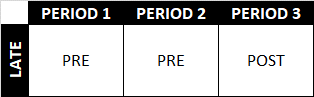 | $logit\left[ P\left( y_{ijk}=Inappropriate \right) \right]=\beta_{0}+\beta_{1}\times Post Intervention_{ijk}$ |
| Model 4 | 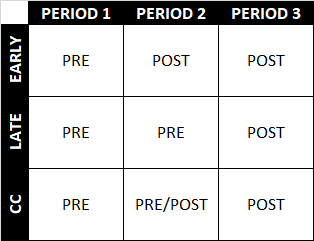 | $logit\left[ P\left( y_{ijk}=Inappropriate \right) \right]=\beta_{0}+\beta_{1}\times Period 2_{ijk}+\beta_{2}\times Period 3_{ijk}+ \beta_{3}\times Early Cohort_{ijk}+ \beta_{4}\times Late Cohort_{ijk}+\beta_{5}\times Period 2_{ijk}\times Early Cohort_{ijk}+\beta_{6}\times Period 3_{ijk}\times Early Cohort_{ijk}+ \beta_{7}\times Period 2_{ijk}\times Late Cohort_{ijk}+ \beta_{8}\times Period 3_{ijk}\times Late Cohort_{ijk}$ |
| Model 5 | 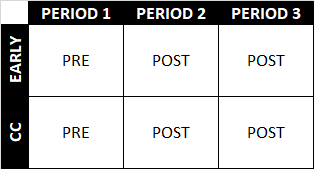 | $logit\left[ P\left( y_{ijk}=Inappropriate \right) \right]=\beta_{0}+\beta_{1}\times Post Intervention_{ijk}+ \beta_{2}\times Early Cohort_{ijk}+\beta_{3}\times Post Intervention_{ijk}\times Early Cohort_{ijk}$ |
| Model 6 | 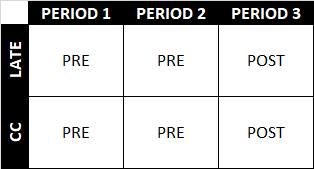 | $logit\left[ P\left( y_{ijk}=Inappropriate \right) \right]=\beta_{0}+\beta_{1}\times Post Intervention_{ijk}+ \beta_{2}\times Late Cohort_{ijk}+\beta_{3}\times Post Intervention_{ijk}\times Late Cohort_{ijk}$ |
| Model 7* | 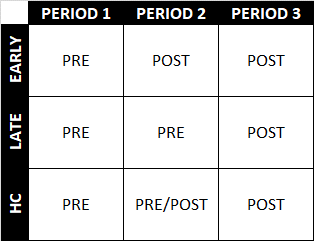 | $logit\left[ P\left( y_{ijk}=Inappropriate \right) \right]=\beta_{0}+\beta_{1}\times Period 2_{ijk}+\beta_{2}\times Period 3_{ijk}+ \beta_{3}\times Early Cohort_{ijk}+ \beta_{4}\times Late Cohort_{ijk}+\beta_{5}\times Period 2_{ijk}\times Early Cohort_{ijk}+\beta_{6}\times Period 3_{ijk}\times Early Cohort_{ijk}+ \beta_{7}\times Period 2_{ijk}\times Late Cohort_{ijk}+ \beta_{8}\times Period 3_{ijk}\times Late Cohort_{ijk}$ |
| Model 8* | 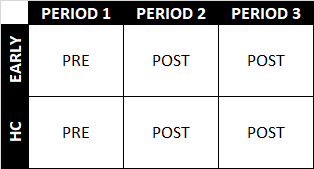 | $logit\left[ P\left( y_{ijk}=Inappropriate \right) \right]=\beta_{0}+\beta_{1}\times Post Intervention_{ijk}+ \beta_{2}\times Early Cohort_{ijk}+\beta_{3}\times Post Intervention_{ijk}\times Early Cohort_{ijk}$ |
| Model 9* | 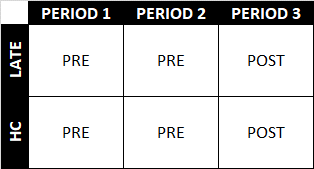 | $logit\left[ P\left( y_{ijk}=Inappropriate \right) \right]=\beta_{0}+\beta_{1}\times Post Intervention_{ijk}+ \beta_{2}\times Late Cohort_{ijk}+\beta_{3}\times Post Intervention_{ijk}\times Late Cohort_{ijk}$ |

CC = Contemporary controls; HC = Historic Controls.

Period 1 is 7/1/14-8/31/14, Period 2 is 9/1/14-2/28/15, Period 3 is 3/1/15-12/31/15, * except for the Historic controls which are Period 1 is 7/1/13-8/31/13, Period 2 is 9/1/13-2/28/14, Period 3 is 3/1/14-12/31/14.

Early and late intervention groups and the historic controls are internal medicine residents only. The contemporary control group included residents and attendings from other specialties.
